# Developing agent-based models of complex health behaviour

**DOI:** 10.1016/j.healthplace.2018.08.022

**Published:** 2018-11

**Authors:** Jennifer Badham, Edmund Chattoe-Brown, Nigel Gilbert, Zaid Chalabi, Frank Kee, Ruth F. Hunter

**Affiliations:** a UKCRC Centre of Excellence for Public Health Research Northern Ireland, Queen's University Belfast, Institute of Clinical Sciences Block B, Royal Victoria Hospital, Belfast BT12 6BA, United Kingdom; b School of Media, Communication and Sociology, University of Leicester, Bankfield House, 132 New Walk, Leicester LE1 7JA, United Kingdom; c Centre for Research in Social Simulation, Department of Sociology, University of Surrey, Guildford GU 2 7XH, United Kingdom; d Department of Social and Environmental Health Research, London School of Hygiene & Tropical Medicine, 15–17 Tavistock Place, London WC1H 9SH, United Kingdom

**Keywords:** Agent-based modelling, Health behaviour, Complex systems, Spatial modelling, Modelling non-communicable diseases

## Abstract

Managing non-communicable diseases requires policy makers to adopt a whole systems perspective that adequately represents the complex causal architecture of human behaviour. Agent-based modelling is a computational method to understand the behaviour of complex systems by simulating the actions of entities within the system, including the way these individuals influence and are influenced by their physical and social environment. The potential benefits of this method have led to several calls for greater use in public health research. We discuss three challenges facing potential modellers: model specification, obtaining required data, and developing good practices. We also present steps to assist researchers to meet these challenges and implement their agent-based model.

## Highlights

• ABM is effective for modelling health behaviour embedded within the environment.
• Key challenges are: formulating rules, obtaining process data, and acquiring skills.
• Experience from other disciplines may be adapted for public health research.
• The potential benefits of ABM warrant the effort required.

## 1 Introduction

Agent-based modelling (ABM) is a computational method that simulates individuals making decisions according to programmable rules. Those rules are set by the modeller to represent key elements of the real world decisions, including the individuals’ own characteristics and their social and physical environment (Bonabeau, 2002, Epstein, 2006, Gilbert, 2008, Railsback and Grimm, 2011). This makes it particularly valuable where place is an important factor in behaviour. There have been several calls for greater use of ABM to understand public health issues and to formulate and evaluate plans to address them (including Auchincloss and Diez Roux, 2008, El-Sayed et al., 2012, Chalabi and Lorenc, 2013). These calls are consistent with broader encouragement of a complex systems perspective of public health issues (Luke and Stamatakis, 2012, Academy of Medical Sciences, 2016, Rutter et al., 2017).

This paper is aimed at public health researchers who have been persuaded by these calls to action and are considering their next steps. It is intended to assist potential modellers to assess whether ABM is a viable and useful method for their research question and set them on an appropriate path if the answer is ‘yes’.

We start by describing relevant features of ABM, emphasising the particular way of thinking that is embodied in the method and the benefits of that framing. The paper then discusses three challenges that are particularly salient for public health researchers who wish to represent human behaviour in ABMs, such as researchers interested in non-communicable diseases, and how these challenges might be overcome. These challenges are: appropriately representing behaviour mechanisms, obtaining data to calibrate those mechanisms and validate the model, and developing the skills to undertake and report ABM based research.

## 2 Agent-based modelling: what and why?

Many issues in public health are complex; that is, behaviour of the system arises partly from interactions rather than simply the characteristics of the individuals within the system (Luke and Stamatakis, 2012, Rutter et al., 2017). Complex interactions can be conceptualised as social processes such as social influence and social support (Berkman et al., 2000), and as place effects such as air quality and transport availability (Macintyre et al., 2002). Complex systems also involve interactions through time, where actions in the past affect the future decision making context; for example the feedback cycle (presented in Rutter et al., 2017) where a smoking ban in public areas reduces the visibility of smoking, which reduces uptake and hence future visibility.

Models are used to help understand, interpret and forecast system behaviour. However, traditional modelling methods focus on individuals rather than their interactions and are therefore not well suited to understanding complex systems or characterising their future behaviour (Smith and Conrey, 2007, Resnicow and Page, 2008, Luke and Stamatakis, 2012, Rutter et al., 2017). Even systems with simple entities and interactions can lead to behaviour that cannot be understood and analysed from the assumption of independent individuals. Instead, complex systems methods such as system dynamics, social network analysis and agent-based modelling explicitly model interactions, directly representing some theoretical understanding of their real world existence and effects (Gilbert and Troitzsch, 2005, Luke and Stamatakis, 2012, Badham, 2014, Sayama, 2015).

In an ABM, simulated individuals make decisions according to programmed rules. What is distinctive about ABM is that the representation is agent-centric (to use the terminology of Wilensky and Rand, 2015): the rules represent the process or mechanism by which the simulated individuals make their decisions, including their personal characteristics and the social and physical environment. That is, causation is expressed directly in model rules as ‘I, the agent, have certain characteristics and beliefs of my own as well as information about the world around me, and therefore will do some action’ (see examples below). Those actions may affect the agent's characteristics (such as adopting some behaviour) and may also influence the agent's environment, for example by consuming resources.

Agent-centric representation allows ABM to deal with interaction and change because the behaviour of the system is generated by (or emerges from) the actions of the simulated individuals and is measured from the simulation output (Gilbert, 2008, Chalabi and Lorenc, 2013, Spruijt-Metz et al., 2015). The model is ‘run’ by stepping through simulated time with agents remaking their decisions. Both agent-agent and agent-environment interactions are expressed in the rules. Agents adapt over time by changing their decisions as the situation around them changes. Heterogeneity is also accommodated, as the same agent in different situations can make different decisions, and different agents in the same situation can make different decisions.

ABMs therefore allow potentially greater fidelity between the complex system being modelled and the model. In turn, this fidelity supports extrapolation from model behaviour to real world system behaviour, which allows insights from the model to be used to understand the system and compare policy options.

In public health, ABM is particularly suited to infectious disease epidemiology, where interactions between individuals are a key driver of system behaviour and the transmission mechanisms are relatively well understood. There are several large, established ABM epidemic models to project epidemic impact under hypothetical outbreak control options (Eubank et al., 2004, Van den Broeck et al., 2011, Grefenstette et al., 2013). There are also detailed models of specific diseases in specific locations (Hunter et al., 2017).

In contrast, there is limited use of ABM in non-communicable disease research. A recent review (Nianogo and Arah, 2015) identified only 22 studies. Furthermore, six of these studies simply use the language and tools of ABM to conduct simulations of independent, individual-based processes (such as disease progression) over a heterogeneous population, but do not include interactions. Such use of ABM is outside the scope of this paper as the systems being modelled are not complex and other methods are available, such as microsimulation or Markov models (Weinstein et al., 2003).

This difference in activity raises the question as to why ABM is not more popular in non-communicable disease research, particularly since the social and physical environments are known to influence many health behaviours (Macintyre et al., 2002). In this paper, we argue that there are three salient challenges in ABM for the potential modeller of non-communicable diseases.

We first describe two public health ABMs, to clarify the benefits of the perspective provided by this method and to assist with the discussion of challenges. These examples were selected primarily because of the published level of detail about agents’ decision rules and source data. Both models focus on human behaviour, but in different public health contexts (active travel and protective behaviour). Both also explicitly model place, and the spatial factors influence the behaviour of the agents. In addition, the models have different purposes and hence level of detail in their representation of real-world behaviour.

### 2.1 Two example agent-based models

The ABM by Yang and Diez-Roux (2013) simulates decisions about whether children will walk to school based on perceived safety and the distance to be walked (see Box 1 for summary). The model objective is to generate hypotheses for later research. Consistent with the objective of plausibility rather than realism, much of the model design has an intuitive connection to the real world rather than representing a detailed theoretical behaviour mechanism. For example, it is reasonable that areas with more walkers are perceived to be safer, but it is less clear that the risk of a traffic accident is the main concern. There are many potentially important factors that do not appear in these rules at all, but that may be required for a more realistic model, such as the presence of major road crossings on the route to school, the age and maturity of the child, and the weather. Other abstractions in the model include the regular layout of streets, central location of schools and arbitrary uniform distributions used to allocate attitude and concern values for agents.

Box 1 **Key features of the walking to school ABM by Yang and Diez-Roux (2013).**

Example ABM: Walking to school

**Modelled process:** Households making decisions about whether their child should walk to school.

**Purpose:** To generate hypotheses for later research, particularly concerning safety interventions and school placement.

**Reference:** Yang and Diez-Roux (2013)

**Process specification:** Agents take into account the household's attitude toward walking to school and two barriers of known importance: perceived safety and distance to school. This is expressed in two conditions (adapted from Eqs. 1–4 of Yang and Diez-Roux, 2013). Whether the child is willing to walk (Eq. (1)) combines the child's attitude (*A*) and the distance to be travelled (*d*, with a decay parameter β). Whether the child's household allows the child to walk (Eq. (2)) assesses whether there are sufficient walkers (*W*) on the path that the child would take to satisfy the concern (*C*) of the child's household about safety. If both conditions are met, the child walks.

(1) $$A+e^{-\beta d}>1$$

(2) $$\mathrm{mean}\;\mathrm{over}\;\mathrm{route}\left(1-W^{-0.6}\right)>C$$

**Agent characteristics:** Attitude and concern level are personal attributes of the agents. Concern is fixed over time. Attitude changes in response to changes in the total number of children walking, which provides a ‘safety in numbers’ feedback cycle over time; more walkers increases attitudes and safety, which both tend to increase the number of walkers. The physical environment influence is expressed through the distance element. The social environment is represented through the number of walkers on the specific route to be taken by the child.

**Calibration:** Attitude and concern level are randomly drawn from an arbitrary uniform distribution for each household. Some parameters were set from theory; $W^{-0.6}$ is the probability of a pedestrian-car collision from prior research. Other parameter values were assigned to give the best fit between model estimates of the proportion of children walking different distances and travel survey data.

**Validation:** Quality of the fit concerning proportion of children walking by distance and travel survey data.

The model was used to compare the effect on the number of walkers of scenarios such as different school location, population density, or allocation of resources for improving safety. Model outputs suggested that more intensive safety improvements near schools may have a greater impact than smaller safety improvements over a larger area. This would need to be tested experimentally before any policy decisions could be taken, but such experimentation could simultaneously support model refinement as more information became available about real world behaviour.

The TELL ME model (Badham and Gilbert, 2015) simulates decisions by individuals to adopt (or drop) protective behaviour in response to an influenza epidemic. Those decisions are based on their own attitude, the proportion of nearby agents who are protected and the proximity of new infections (see Box 2 for summary). It was intended to be sufficiently realistic to compare options about communication plans, and support decisions without the need for further research. A way to model different communication plans and the response to communication were also built into model rules.

Box 2 **Key features of the TELL ME ABM of protective behaviour in response to an influenza epidemic by Badham and Gilbert (2015).**

Example ABM: Epidemic-protective behaviour (TELL ME)

**Modelled process:** Individuals making decisions about whether to adopt protective behaviour such as vaccination or increased hand washing in response to an influenza epidemic.

**Purpose:** To (assess the feasibility of using ABM to) compare options about different types of government communication to encourage protective behaviour, so as to support decision making.

**References:** Badham and Gilbert (2015), TELL ME (2015)

**Process specification:** The agent decides whether to adopt or drop protective behaviour based on three factors (at Eq. (3)): the agent's own attitude (*A*), perceived norm (*N*) and epidemic risk. Norm is the proportion of nearby individuals who have adopted protective behaviours. Epidemic risk is the discounted sum of the epidemic incidence (*I*) in nearby locations. Behaviour depends on whether the weighted sum of these factors exceeds the threshold (*T*).

(3) $$\omega_{A}A+\omega_{N}N+\left(1-\omega_{A}-\omega_{N}\right)\overset{t}{\underset{j=0}{\sum }}\delta^{t-j}I_{t-j}>T$$

The model allowed communication to be described by features such as medium (e.g. mass media or social media), timing, and target population. Targeted agents exposed to the communication adjust their attitude or perception of norms (that is, increased *A* and *N* in Eq. (3)) or adopt the behaviour temporarily regardless of their attitude and situation.

**Agent characteristics:** Attitude is a personal characteristic. Weights ($\omega_{A}$, $\omega_{N}$), incidence discount (δ) and threshold (*T*) are identical for all agents. Exposure to communication is randomly assigned based on probabilities set by the model user.

**Calibration:** Population density was drawn from national statistics. Disease progression variables were set from influenza progression literature and travel surveys. Attitude was randomly drawn from a distribution based on hand hygiene attitude surveys. Weights and threshold were set to give the best fit between model estimates and time-series data of protective behaviour during an epidemic. Communication parameters were not calibrated.

**Validation:** Quality of fit to the pattern of protective behaviour. Workshops with experts also assessed realism of model outputs.

Ultimately, the TELL ME model was not suitable for detailed communication planning because there was insufficient understanding of the behaviour being modelled to support that purpose, and insufficient data to overcome that gap. However, it was suitable for purposes that are less demanding of realism, such as education and exposing data needs (Barbrook-Johnson et al., 2017).

### 2.2 Benefits of agent-based modelling

Before developing our argument about challenges, we draw from the two examples to clarify how the agent-centric representation differs from other modelling methods and the benefits of this approach. Regression and other statistical approaches attempt to describe the relationship between aggregate variables (e.g. the proportion of people adopting protective behaviour and epidemic incidence), relying on the pattern in any data rather than specifying the causal connections between the variables. System dynamics (Sterman, 2000, Homer and Hirsch, 2006), another common complex systems modelling method, is able to represent interactions but also operates with aggregate variables and the interactions are implemented at the macro level. In contrast, as already described, an ABM directly describes causal processes and generates the overall system behaviour, capturing interactions over time between agent behaviour, other agents and the environment.

This approach allows ABMs to effectively model systems governed predominantly by micro-level interactions or where there is substantial heterogeneity in agents’ characteristics or their environment. Such capacity is the key benefit of ABM, matching the assumptions of the modelling method to the drivers of the real world system behaviour.

In both presented examples, micro-level interactions between individuals operate through place. In the walking to school model, the presence of other walkers on the walking route contributes to the measure of safety. In the TELL ME model, agents directly influence each other's decisions because the decision rule includes the proportion of nearby individuals who have already adopted protective behaviour. ABMs are also able to model direct interaction such as transmitting infections or information from one individual to another.

Both examples also included interactions between individuals and their physical environment. In the walking to school model, that environment was represented in a very abstract way with a fixed grid of roads and central location of schools. The distance to walk was a key factor in the walking decisions. In contrast, the TELL ME model uses GIS tagged population density data to transmit the simulated epidemic. This supports mutual interaction between the individuals and their environment; proximity to new infections influences agent adoption of protective behaviour, and agents adopting protective behaviour reduce the epidemic incidence in their location. ABMs are able to incorporate increasingly sophisticated spatio-temporal observational data (Eubank et al., 2004, Van den Broeck et al., 2011, Tompkins and McCreesh, 2016, Lange and Thulke, 2017).

Heterogeneity is expressed in both environment and agent characteristics. For example, the households in the walking to school model had varying attitudes and thresholds for concern, as well as distances to school. The combination of personal and situational factors generated different household behaviours despite the same decision rule being applied for each.

Both example ABMs were developed to compare alternative hypothetical policy options, a common use of models. In the public health context, pre-testing policy options allows the limited time and resources available for trials to be targeted to those interventions expected to deliver the greatest benefit (Auchincloss and Diez Roux, 2008, El-Sayed et al., 2012). However, the generation of system behaviour has additional benefits in complex systems, where behaviour can be counterintuitive and difficult to connect to the underlying mechanism. Running the model can perform an education and communication role (as proposed for TELL ME in Barbrook-Johnson et al., 2017). ABMs can also assist with theory development by demonstrating whether a proposed mechanism provides a plausible explanation of some observed behaviour, or to compare competing explanations (Smith and Conrey, 2007, Chalabi and Lorenc, 2013, Chattoe-Brown, 2013).

The agent-centric representation of ABMs and the focus on decision making mechanisms delivers benefits above the capacity to model complex systems. Rules express behaviour in a natural, albeit abstract, way and it is relatively straightforward to represent social and physical environments once the relevant aspects have been identified (Bonabeau, 2002, Auchincloss and Diez Roux, 2008). Different types of data may be expressed with those rules, combining expert opinion, quantitative data and qualitative information (Smajgl et al., 2011, Chattoe-Brown, 2013). Further, the clear link between theoretical processes and model rules facilitates community or interdisciplinary engagement (Voinov and Bousquet, 2010). These benefits have been exploited particularly effectively in a series of projects in developing countries to support negotiation in land use or water rights. One of those projects (D'Aquino et al., 2003) assisted Senegalese villagers to develop a role playing game that captured the tension between crop and animal land use, subsequently converted to an ABM to compare proposed policy options for collective regional planning.

## 3 Specification: what to include in the model

The first challenge faced in developing an ABM is model design or specification: expressing the real world behaviour to be modelled in the form of a set of rules. This includes determining the process(es) to be represented and deciding an appropriate level of detail for the purposes of the model, and formulating the process(es) as ‘rules’ to be later translated into code.

While this challenge arises for all ABMs, it is more difficult where there is little theoretical agreement, as is the case for human behaviour (Michie et al., 2014, Jager, 2017). An epidemic model might have human behaviour as an input, such as a model parameter that specifies the reduction in contacts with others when a person becomes infected, but the fundamental processes concern disease transmission. However, for non-communicable disease research, such as estimating the impact of a proposed intervention on human behaviour, that behaviour is the subject of the model and must be generated by the model rules. For example, how much more likely is someone to walk instead of drive for some specified improvement in walkability? The agent-centric process-driven perspective of ABMs requires the mechanisms to be specified even where they are not fully understood.

ABMs have been broadly characterised as theoretical or applied (Bianchi and Squazzoni, 2015, Bruch and Atwell, 2015, DeAngelis and Grimm, 2014, OSullivan et al., 2015, with various terminology). This dichotomy is largely artificial and in practice the amount of real-world detail represented in an ABM falls on a continuum. The position on that continuum is determined by the model purpose, with greater detail required where realism is important, such as assessing options in a specific situation or other applied purposes (Bruch and Atwell, 2015). This can be seen in the two presented examples: the TELL ME model required substantial detail because it was intended to provide specific guidance, whereas a relatively abstract set of rules was adequate in the walking to school model to generate plausible hypotheses for further research.

The challenge is typically expressed as a question of balance, finding relatively simple rules that encapsulate the key theoretical question while also generating the system behaviour of interest (Auchincloss and Diez Roux, 2008, El-Sayed et al., 2012, OSullivan et al., 2015, Smith and Conrey, 2007). Only the most important aspects of the real world are included in the model (abstraction) and those aspects are expressed with simple rules (idealisation). Unfortunately, there is no abstraction and idealisation recipe to follow to guarantee a good set of rules. While the emphasis on model design is typically on making the model rules as simple as possible, it is equally important to ensure they are as descriptive as required to meet the purposes of the model (Edmonds and Moss, 2005).

It is tempting to include too much detail in a model in an attempt to represent the subtleties of expert disciplinary knowledge. This occurs when the model is oriented to what is known instead of the research question and model purpose. Too much detail is counter-productive because it obscures the relationship between the agent and system behaviours. On the other hand, behaviour change theories may require additional detail to be suitable for simulation, either because the theory is not fully specified or to take account of dynamic feedback between psychosocial constructs (Navarro-Barrientos et al., 2011, Riley et al., 2016, Jager, 2017).

Once the relevant factors influencing a process have been identified, each must be elaborated to describe how the personal characteristics of the agent and the social and environmental features of the situation determine the action to be taken by the agent. How these factors change also needs to be specified. In both ABMs described, the rules were expressed as equations with thresholds, but other forms are possible. For example, in their classic model of ethnocentrism, Hammond and Axelrod (2006) used simple if-then statements to decide whether an agent cooperates or defects in a series of Prisoner's Dilemma games with other agents, such as cooperating if the other agent is from the same group.

## 4 Appropriate data: calibration and validation

The second major challenge is to obtain relevant data to calibrate and validate the model (Auchincloss and Diez Roux, 2008). Public health researchers typically have access to extensive cross-sectional data including demographic and other personal characteristics that can be used to describe the heterogeneity of the agents. These data may also include information to characterise relevant attitude and behavioural distributions. Nevertheless, these data may not be suitable for calibrating process rules (that is, setting parameters to appropriate values).

For theoretical models, only limited (or no) data may be required if the objective is to explore some fundamental process. In the walking to school model (Yang and Diez-Roux, 2013), attitude (*A* at Eq. (1)) is assigned from an arbitrary distribution, and the relationship between distance and walking to school (product βd at Eq. (1)) is set so as to best approximate five data points from travel surveys reporting distance ranges and the proportion walking. These data are not process oriented; there is no attempt to establish real world values for the inputs to the process (concern thresholds and attitudes) or to test whether attitude changes are reasonable. As an explicitly exploratory model, however, no more than plausibility is expected.

However, empirical models require extensive data about the process and its measurable outcomes because detailed model results are only meaningful where the detailed behaviour mechanisms have been calibrated. This requires repeated measures of mechanisms (Bruch and Atwell, 2015). Further, an ABM is of most value where situational influences are included, and this imposes the additional requirement for data that reports the relevant social and environmental features.

The TELL ME model, for example, had many detailed rules about the relationships between media messages, attitudes, epidemic risk and protective behaviour (Badham and Gilbert, 2015), and each required similarly detailed data for calibration. Process data were available for modelling the behaviour; inputs (such as attitude and incidence) and outputs (behaviour adoption) were collected in 13 waves of Hong Kong surveys about hand washing and social distancing behaviours (Cowling et al., 2010, TELL ME, 2015), and incidence was reported in official statistics. However, similar data were not available for responses to communication; evaluations of communication campaigns did not distinguish between the effects of specific media elements nor estimate behaviour in the absence of the campaign.

While time-series data enables statistical analysis to calibrate some ABM parameters, other forms of evidence are also useful and may be more readily available. Qualitative methods (interviews and observation), specific experiments (natural experiments, field experiments and role playing games) and expert advice (including participatory methods) can all be used to constrain parameter values to plausible ranges (Smajgl et al., 2011, An, 2012, Chalabi and Lorenc, 2013). The potential to combine information from different sources is one of the strengths of ABM (Chattoe-Brown, 2013). In addition, public health studies are starting to use methods that intentionally collect process-oriented data such as Ecological Momentary Assessment (Moskowitz and Young, 2006) and Just in Time Adaptive Interventions (Nahum-Shani et al., 2014).

It may also be possible to impute the detailed parameter values from other behavioural data. For example, Zhang et al. (2015) use observed changes in relationships and behaviour to fit a model to estimate social network influence, and then use the fitted values to project the outcome under different network scenarios. Pattern-oriented modelling (Grimm et al., 1996) takes a different approach; the model is run with different values in each variable's plausible range and the simulation results are compared to features of the empirical data that are as different as possible. Parameter values are selected that generate system level behaviour that adequately replicates all features.

Validation imposes additional data requirements. Generally, validation of ABMs may be more problematic than for other modelling techniques because the interactions between model entities yield complex effects on overall system behaviour and both quantitative and qualitative data are used to inform their development (Windrum et al., 2007, Gilbert, 2008). Errors associated with misspecification of the casual mechanisms can therefore be difficult to detect (Murray et al., 2017). Some of the validation methods used are more subjective than those used in other systems modelling techniques such as differential equations, discrete-event simulation, microsimulation and Markov models.

Regardless of the form of validation, the additional data must be independent of calibration data (rather than simply a subset) to avoid simply affirming the consequent (El-Sayed et al., 2012) and may be at a different level of detail. ABM does have one validation advantage however. The multi-level representation can be utilised by calibrating parameter values at the micro-level (of processes and relationships), and observing the macro-behaviour of the ABM to assess the realism of overall system behaviour.

## 5 Developing good ABM practices

The final significant challenge for public health researchers who wish to use ABM is developing the necessary skills (Smith and Conrey, 2007, Luke and Stamatakis, 2012). This is not simply the skills for actually building the model, but also knowledge of good practice for experimenting with it and analysing and reporting the results.

A public health researcher who wishes to use a novel statistical technique is able to run it within a familiar software environment, read documentation to determine appropriate syntax, and draw on extensive statistical training and common output formats to interpret the results. There may also be colleagues available to provide assistance who have previously implemented the technique, or at least have greater relevant statistical experience.

In contrast, a public health researcher who wishes to use ABM has limited (if any) relevant background in their professional training, and few examples or experienced colleagues to learn from. While guides of varying detail (Smith and Conrey, 2007, Railsback and Grimm, 2011, Wilensky and Rand, 2015) and short courses are available, it can be difficult to adapt the learning to a specific research question without ongoing support.

An ABM is developed in the same way as other computer programs. While there are specific ABM software platforms to make the task easier, public health researchers do not typically have programming training. The difficulty is not just a lack of knowledge of the language, but experience is required to write code that is both efficient and readable.

Working with a programmer is the obvious way of obtaining programming skills for a public health ABM project. However, it is not simply a matter of delegating the ABM programming, because designing the model requires both agent-centric thinking and public health subject matter expertise, so collaboration is important. A ‘team science’ approach is therefore more appropriate, ideally moving toward development of interdisciplinary researchers (Academy of Medical Sciences, 2016). It may also be beneficial for the subject matter expert to learn some ABM programming to better understand the agent-centric perspective.

The most popular ABM programming language is NetLogo (Wilensky, 1999, Janssen, 2017), which is powerful, flexible and relatively easy to learn. For public health researchers intending to develop the model themselves, NetLogo has the additional advantages of being self-contained (no other language requirements) and is the language typically demonstrated or taught in introductory ABM courses and textbooks (including Railsback and Grimm, 2011, Wilensky and Rand, 2015). Experienced programmers are likely to prefer ABM tools that integrate with their preferred language, such as RePast for java or Mesa for python. Language choice may also be constrained by the desired functionality of the model, such as a very large number of agents, complex GIS integration or specific visualisation needs.

Having designed and constructed a model, the researcher is also faced with questions about how to use it (such as selecting parameter values to test and the number of simulation runs for each combination) and what to report in journal article(s). While ABM is a novel method in much of public health, it is an established, albeit relatively uncommon, method in other disciplines. Suitable co-researchers and case studies may be available in sociology, where many models are more theoretical (Bianchi and Squazzoni, 2015), or in ecology, which has a strong tradition of applied models (DeAngelis and Grimm, 2014). Potential co-researchers may also be identified from consideration of key elements to be modelled such as spatial considerations (geography, see Heppenstall et al., 2011) or population structure (demography, see Silverman, 2018).

Sensitivity analysis is a critical practice in ABM (Saltelli and Annoni, 2010, Railsback and Grimm, 2011) to understand the limitations of any results, and can also ameliorate the effect of inadequate data. Simple sensitivity analysis systematically varies the model inputs and analyses the impact on the simulation results (for example, a 10% increase in an input may contribute to a 5% decrease in an output).

With complex systems, the effect of interactions means it is not sufficient to vary each input separately. However, ABMs typically have many input parameters and it may not be feasible to run simulations for each combination of varied input values. Instead, more sophisticated methods are required that efficiently sample the input parameter space, for example Latin Hypercube sampling or sets of random walks (Saltelli and Annoni, 2010). For very large ABMs that take substantial running time, the many simulations required for sensitivity analysis may instead be performed on a statistical model that emulates the ABM (OHagan, 2006, Wang and Shan, 2007).

Good practice guidelines are available for other aspects of the modelling process such as experimental design (Schmolke et al., 2010, Lorscheid et al., 2012) and documentation (Grimm et al., 2010, Dorin and Geard, 2014), although they are not always followed even in disciplines where the use of ABM is more mature (Angus and Hassani-Mahmooei, 2015, Janssen, 2017). At a minimum, it is essential that documentation describes the decision rules, parameter values and their source, and the number of simulations performed. However, implementing good documentation practices may be constrained by the publication process in public health, with page limits and standard headings that are not appropriate for ABM based research, and only a small pool of reviewers with relevant expertise (Rutter et al., 2017).

## 6 Discussion and conclusion

Models are used to understand systems, communicate theory, shape intervention design, compare policy options, and for many other purposes. The agent-centric representation of ABM corresponds with the mechanisms of complex system behaviour driven by interactions between heterogeneous individuals and with their environment. This makes it particularly appropriate for modelling social and place effects in public health.

ABM is a mature methodology in many disciplines that concern the relationship between people's behaviour and their environment. While it has become established in communicable disease epidemiology, it is relatively uncommon in other areas of public health. This paper describes three salient challenges for ABM where human behaviour is to be simulated, such as non-communicable disease research.

The first challenge is to express real world behaviour as a set of appropriately detailed agent-centric rules. The agent-centric perspective must take into account the relevant characteristics of the people making decisions and the social and physical environment that influences that decision. The level of detail must allow the model to generate behaviour that is sufficiently realistic to provide insight into the research question. The second challenge is to obtain the process oriented data necessary to calibrate the model rules and validate the model as fit for purpose. The final challenge is to acquire the specific skills necessary to develop an ABM and the knowledge to adopt good practice.

Before embarking on a project to develop an ABM, it is useful to consider whether ABM is the most appropriate methodology. The questions and comments provided at Box 3 are intended to stimulate such a consideration. While it is not necessary to answer all questions in advance, reflecting on potential responses can assist in overcoming the identified challenges.

Box 3 **Questions to consider in the planning phase of an agent-based modelling research project.**

**What is the purpose of the model?** Possible responses include generating hypotheses for further research, comparing the effect of different potential policy options, integrating information from different sources concerning an issue, testing the plausibility of some causal claim, and engaging stakeholders in developing a common understanding of an issue. Two follow-up questions are: How much detail is required to meet this purpose, and what is the process to be modelled?

**Is agent-centric representation the most appropriate?** The benefits of ABM arise from its natural representation of processes that involve interactions and heterogeneity. There are other modelling techniques available if the actions of one person (or other entities to be modelled) do not influence the actions of others, or if entities can be meaningfully represented by their average.

**Is the process to be modelled understood at an appropriate level of detail?** Is there a theory about the personal, social and environmental elements that influence a person's decision? How can those elements be combined? Is there a theory about how a person's decision influences others, impacts on the environment, and influences their own future decisions?

**Are there (quantitative or qualitative) data to calibrate at the appropriate level of detail?** Are there data about the distribution of personal characteristics that are to be included in the decision process? Are there data concerning all of the relevant environmental factors in the process?

**Can the model be validated to a standard adequate for the purpose?** Are data available in addition to those to be used for calibration, perhaps describing the system at a different level? Are there characteristic patterns in the system behaviour that would be expected in the simulation?

**Who is to develop the model?** How is that person to develop programming skills or subject matter knowledge if required? How is the model developer to obtain advice on technical and subject matter issues?

**How can good practices in experimentation, documentation, and reporting of results be implemented?** Are there exemplar models available from public health or other disciplines? Is the modeller (and other members of the research team) familiar with practice guidelines?

It is also important to recognise that extending ABM to new areas will necessarily be gradual. While a great deal can be learned from other disciplines, experience provides its own insight. Even unsuccessful modelling projects are valuable, for example by highlighting gaps in our understanding of mechanism or in data (Auchincloss and Diez Roux, 2008). Research in progress (such as Hennessy et al., 2016) is an important supplement to material about completed ABMs to assist public health researchers to understand how to apply the methodology to their own questions.

This paper describes these challenges not to discourage potential modellers, but to provide pointers to recognised pitfalls and potential paths to avoid them. The agent-centric representation embodied in ABM has substantial promise for understanding the complexity of non-communicable disease and other areas of public health research. This promise makes the effort of building ABMs worthwhile, but public health researchers must work gradually and have appropriate expectations of what may be achieved at each step.
